# Parents’ hope in perinatal and neonatal palliative care: a scoping review

**DOI:** 10.1186/s12904-023-01324-z

**Published:** 2023-12-18

**Authors:** Aline Oliveira Silveira, Monika Wernet, Larissa Fernandes Franco, Patrícia Luciana Moreira Dias, Zaida Charepe

**Affiliations:** 1 Faculty of Health Sciences, Graduate Program in Nursing, University of Brasília (UnB), Brasilia, Federal District Brazil; 2 Biological and Health Sciences Center, Graduate Program in Nursing, Federal University of São Carlos (UFSCar), São Carlos, São Paulo Brazil; 3 MSc in Health Sciences by the Graduate Program in Nursing, Federal University of São Carlos (UFSCar), São Carlos, São Paulo Brazil; 4 Postdotoral researcher at the Graduate Program in Nursing, Federal University of São Carlos (UFSCar), São Carlos, São Paulo Brazil; 5 Faculty of Health Sciences and Nursing Center for Interdisciplinary Research in Health (CIIS), at Universidade Católica Portuguesa, Lisbon, Portugal

**Keywords:** Hope, Neonatal, Perinatal, Family, Palliative care

## Abstract

**Background:**

The diagnosis of a life-limiting condition of a child in the perinatal or neonatal period is a threat to parental hopes. Hope is an interactional and multidimensional construct, and in palliative care, it is a determinant of quality of life, survival, acceptance and peaceful death.

**Objective:**

To map scientific evidence on parents’ hope in perinatal and neonatal palliative care contexts.

**Method:**

a scoping review theoretically grounded on Dufault and Martocchio’s Framework, following the Joanna Briggs Institute methodological recommendations. Searches were performed until May 2023 in the MEDLINE, CINAHL and PsycINFO databases. The searches returned 1341 studies.

**Results:**

Eligible papers included 27 studies, most of which were carried out in the United States under a phenomenological or literature review approach. The centrality of women’s perspectives in the context of pregnancy and perinatal palliative care was identified. The parental hope experience is articulated in dealing with the uncertainty of information and diagnosis, an approach to which interaction with health professionals is a determinant and potentially distressful element. Hope was identified as one of the determinants of coping and, consequently, linked to autonomy and parenthood. Cognitive and affiliative dimensions were the hope dimensions that predominated in the results, which corresponded to the parents’ ability to formulate realistic goals and meaningful interpersonal relationships, respectively.

**Conclusion:**

Hope is a force capable of guiding parents along the path of uncertainties experienced through the diagnosis of a condition that compromises their child’s life. Health professionals can manage the family’s hope by establishing sensitive therapeutic relationships that focus on the dimension of hope. The need for advanced research and intervention in parental and family hope are some of the points made in this study.

**Protocol registration:**

https://osf.io/u9xr5/.

**Supplementary Information:**

The online version contains supplementary material available at 10.1186/s12904-023-01324-z.

## Background

The discovery of a pregnancy is an essential milestone in the parenting process and creates expectations about life, health and the child [1]. When challenged by prematurity, malformations, congenital diseases and even death, expectations are frustrated, and suffering is experienced [1]. Receiving the diagnosis of a child’s life-threatening condition is challenging to cope with and requires professional support aligned with palliative care (PC).

Perinatal and neonatal Palliative Care is a coordinated care strategy that encompasses actions in obstetric and neonatal care, focusing on maximizing quality of life and comfort for fetuses and newborns with conditions considered life-limiting [2]. The PC practice is not aimed at a disease-modifying treatment but, rather, a collaborative support process for the incorporation and management of losses experienced, centered on people, their values and beliefs [2, 3]. Critical elements in the context of perinatal and neonatal PC are shared decision-making, care planning and coping with distress, with attention to its early introduction and maintenance into childhood [2].

The concept of hope in neonatal and perinatal contexts is related to parental expectations and desires for mobilizing energy to achieve babies’ positive outcomes [4]. Regarding attributes, they are an experiential process targeted toward the present and future in your lives [4]. Hoping is not a single act but a complex of many thoughts, feelings and activities that change with time [5].

Hope is a multidimensional dynamic life force characterized by confidence yet uncertainty about achieving a future good, which is realistically achievable and personally necessary to the hoping person. In this context, hope has implications for action and interpersonal relatedness, which are directly associated with family coping [6, 7]. In this framework, hope is conceptualized as being compared to two spheres (generalized hope and particularized hope) and six dimensions (affective, cognitive, behavioral, affiliative, temporal, and contextual) [5]. Generalized hope has a broad scope and is not linked to any concrete or abstract hope object. It is the same as intangible hope. Particularized hope involves a special effect or being in a good state – a hope object. Hope objects that are hoped for can be concrete or abstract, explicitly stated, or implied [5].

Each of the six dimensions has a set of components that structure the hope experience. Changes in emphasis within and among the dimensions and their elements characterize the process of hoping. In the same instances, multiple hope processes related to different objects are active in the same person at the same time: (i) the **affective dimension** of hope focuses on sensations and emotions that permeate the entire hoping process; (ii) the **cognitive dimension** focuses on the processes by which individuals wish, are imaginative, wonder, perceive, think, remember, learn, generalize, interpret and judge about the identification of hope objects; (iii) the **behavioral dimension** focuses on the hoping person’s action orientation about hope; and (iv) the **affiliative dimension** focuses on the hoping person’s sense of relatedness or involvement beyond the self as it bears upon hope. This dimension includes social interactions, mutuality, attachment and intimacy, other-directedness, and self-transcendence; (v) the **temporal dimension** focuses on the hoping person’s experience of time (past, present, and future) about hopes and hoping. Hope is directed toward a future good, but the past and present are also involved in the hoping process. (vi) The **contextual dimension** of integrated hope is brought to the forefront of awareness and experience within the context of life as interpreted by the hoping person. The contextual dimension focuses on those life situations or circumstances that surround, influence and are part of a person’s expectations and are an opportunity for the hoping function to be activated or to test hope [5].

Parents of children who have life-limiting and life-threatening diseases undergo profound and pervasive uncertainty, leading to their own illness experience being described as a dual reality in which fighting for survival and recognizing the threat of their child’s death are daily challenges. Hope is an indispensable and vital source of strength for parents, allowing them to cope with their children’s condition and with palliative care and enabling them to continue living. Recognizing the meanings, facets and dimensions of hope that emerge in parents’ experiences may help health professionals better understand and develop a comprehensive, collaborative, and supportive care plan that considers the intricacies inherent to the complexity of the parents’ experience and the importance of hope [6, 7]. This allows for alignment with family-centered care.

The present study sought to integrate knowledge and to advance the understanding of hope within the context of neonatal and perinatal PC, expand health interventions focused on hope, and foster new research in the area. The objective was to map scientific evidence on parental hope in the perinatal and neonatal palliative care contexts.

## Methods

**Fig. 1:**
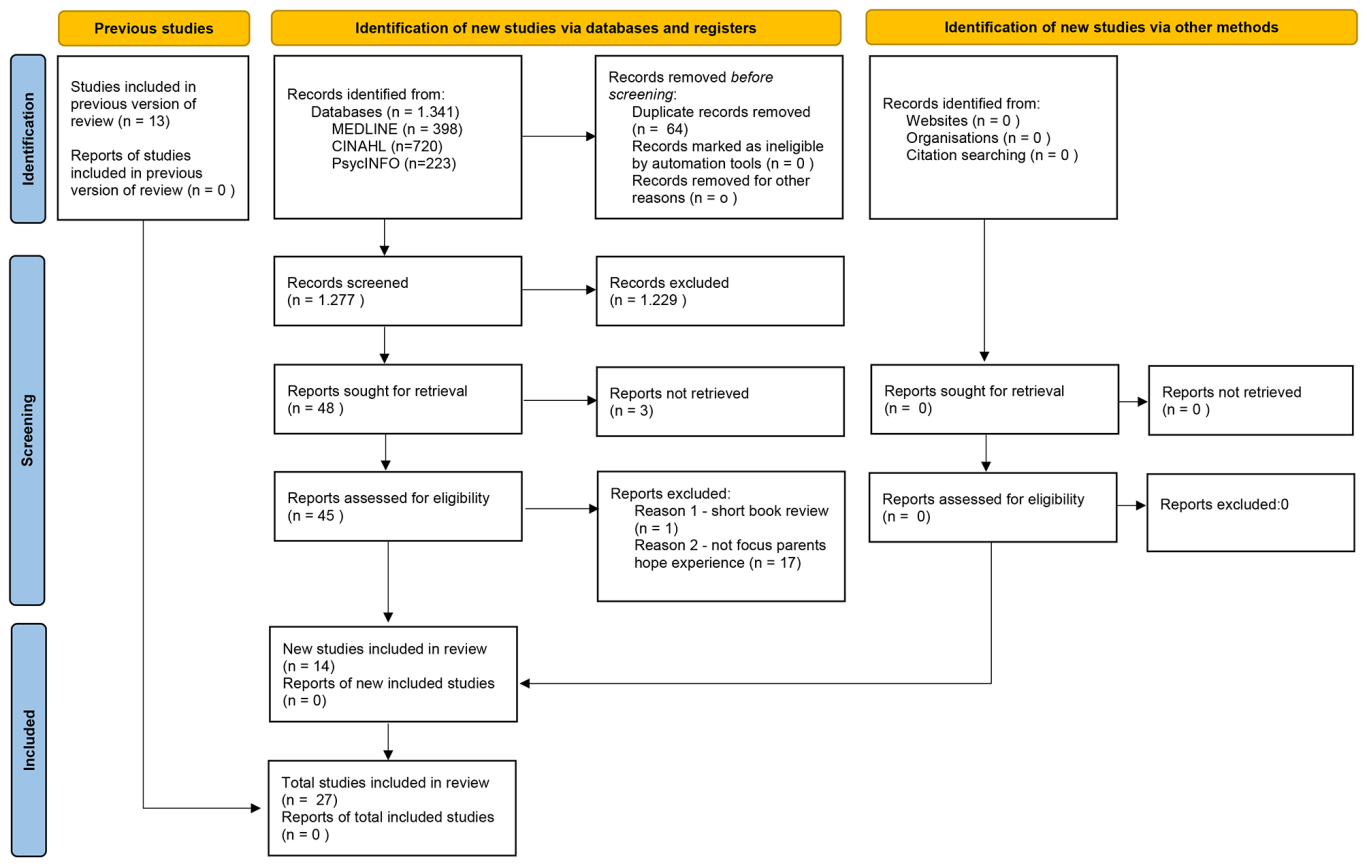
PRISMA flow chart of study selection [7]

This study was developed through the five steps recommended in the Joanna Briggs Institute (JBI) protocol [8]: definition of the research question; identification of relevant studies; selection and inclusion of studies; data organization; and grouping, synthesis, and reporting of results. To ensure the integrity of this manuscript and methodological rigor, the Preferred Reporting Items for Systematic Reviews and Meta-Analyses extension for Scoping Reviews (PRISMA-ScR) checklist and the PRISMA flowchart of the study selection (Fig. 1) were used to report this study [9].

A primary survey of the International Prospective Register of Systematic Reviews (PROSPERO) and The Cochrane Library was carried out in the second half of 2020, with no previous reviews identified. The research protocol was registered and updated in the Open Science Framework on July 26th, 2023 (https://osf.io/u9xr5/).

The PCC mnemonic was used to elaborate the guiding question: Which is the evidence on the hope of parents who experience perinatal and neonatal palliative care? Population in question (P): parents experiencing fetal and neonatal conditions eligible for palliative care; Concept (C): hope [5]; and Context (C): palliative care.

The research was carried out in the following databases: Medical Literature Analysis and Retrieval System Online (MEDLINE), Cumulative Index to Nursing and Allied Health Literature (CINAHL) and American Psychological Association (APA PsycINFO) in February 2021, updated in May 2023. Original studies were selected, such as randomized and nonrandomized clinical trials, bibliographic, documentary, experimental, field and case studies, review studies and reflective or theoretical studies, with the following types of approaches: quantitative, qualitative, theoretical, or mixed-methods studies, primary and secondary (reviews/reflective/theoretical) and data triangulation. There was no restriction regarding the research period, and the languages included were English, Spanish, and Portuguese. Exclusion criteria were applied to publications covering other age groups, articles focusing exclusively on death, and those in which the target audience was health professionals and not parents. Letters to the editor, abstracts, clinical protocols, and annals of scientific events were also excluded.

The databases and the language limits were defined based on pretests, and several preliminary searches were carried out with the support and input of a research librarian. These databases were chosen for being the most appropriate for the scoping review on parental hope in perinatal and neonatal palliative care, as they have the most relevant production on this subject matter. Regarding language, we considered the feasibility criterion; however, notably more than 90% of the production on the topic is in English.

The gray literature was an exclusion criterion for this scoping review. As the intersection between the concepts of parental hope and perinatal and neonatal palliative care is a new and emerging process in terms of science, the decision was made to map the literature published on this topic.

This study considers the WHO definition of pediatric palliative care applied to perinatal and neonatal palliative care [10–12]. Neonatal-Perinatal Palliative Care is a complete, multidisciplinary approach to providing comprehensive care to families when there is a potentially life-limiting, serious or clinically complex fetal or neonatal diagnosis (from 22 gestational weeks to 28 weeks after birth) to relieve pain and control symptoms and improve the care quality and well-being of fetuses and infants, their families and health care providers involved. It is holistic, family-centered, comprehensive, and multidimensional, so it addresses not only the physical aspect but also the psychological, social, and spiritual dimensions [2, 10, 11].

Concerning eligibility criteria, in this study, the definition of populations in need of perinatal and neonatal palliative care was adopted [2, 10, 11]. The criteria were families or parents facing a condition in which there is a lethal diagnosis in the prenatal or neonatal period or a diagnosis for which there is little or no prospect of long-term survival without severe morbidity or extremely poor quality of life and for which there is no cure [2, 10, 11].

The lists of references of the articles selected were systematically searched to find additional literature relevant to this review.

The search strategy adopted was developed with descriptors and Boolean operators, as well as the MeSH descriptors applied in the scientific databases. (for full search strategies, see Supplementary Table 1).

All the studies retrieved were imported into Rayyan.ai reference management software (https://www.rayyan.ai/), and duplicates were removed. Articles were initially selected by examining their titles and abstracts and, in a second selection stage, based on full-text reading. The selection process was performed based on the inclusion and exclusion criteria prespecified in the review protocol. An independent review was carried out by two pairs of reviewers (AOS, MW, LFF, and PLMD) to reach consensus and increase consistency when selecting the articles that were part of this review. This reference manager software was also used to organize the results.

The process of extracting data from the sources of evidence followed the JBI instrument template for source of evidence details, characteristics, and results extraction. In this study, the following information was extracted: author, year of publication, country where the study was conducted, participants, theoretical and methodological framework, evidence of hope, and implications.

The analytical process took place at the level of characterization of the studies and publication trends and at the level of hope evidence in the parental experience of perinatal and neonatal palliative care. The parental hope evidence was extracted in full and grouped into thematic categories representing the hope dimensions (affective, cognitive, behavioral, affiliative, temporal, and contextual) according to the theoretical model guiding this scoping review [5]. In addition, an analysis of the implications of the studies was conducted to map the gaps and challenges for practice and research into parental and family hope in the context of pediatric and neonatal palliative care.

Studies investigating the perspectives of parents and health professionals were included. The results are presented separately, but only the content referring to the parents’ perspective was considered as the *corpus* in the process of analyzing and synthesizing the evidence.

The results are summarized in Table 1 with the characterization of the studies, including title, country, publication date, aim, participants and study design. The hope evidence and corresponding dimensions according to Dufault and Martocchio’s Framework [5] are presented in Table 2.

**Table 1:** Characteristics of the studies included in the review

ID	Country and Publication date	Aim	Participants	Design
S1^12^	United Kingdom 2023	Increase understanding of how parents experience the antenatal diagnosis of a life-limiting or life-threatening condition during pregnancy and following the birth of their baby.	Not applicable	Qualitative meta-ethnography
S2^13^	Netherlands 2023	Provide an insight about parent personal experiences of their sons’ birth and making decisions at the limit of viability.	Not applicable	Reflexive Narrative
S3^14^	Ireland 2023	Explore the care experiences of parents whose pregnancy was diagnosed with fatal fetal anomaly.	6 mothers and 4 fathers of children diagnosed with a fatal fetal anomaly	Qualitative phenomenological study
S4^15^	Germany 2022	Enhance our knowledge about care experiences and needs of parents who decide to continue pregnancy despite the life-limiting condition of their unborn child and to reconstruct their pathway through existing healthcare structures.	Mothers and fathers who continued pregnancy following a severe and life-limiting fetal diagnosis	Qualitative descriptive study
S5^16^	USA 2022	Provide an overview of the role that Perinatal Palliative Care providers play in the care of families facing severe or life-limiting neurologic diagnoses.	Not applicable	Literature Review
S6^17^	USA 2022	Explore the experiences of women who received life-limiting fetal diagnoses during pregnancy and support from a perinatal palliative care program.	12 women who experienced pregnancies with life-limiting fetal diagnoses and received care from a perinatal palliative care program.	Qualitative descriptive study
S7^18^	Brazil 2021	Synthesize qualitative evidence from primary studies to better understand the experience of the spirituality of bereaved parents and its relationship with adapting following a stillbirth.	Not applicable	Qualitative meta-synthesis
S8^19^	USA 2021	Report on what is known about the cultural, spiritual, and religious practices of parents and how this might impact the neonates who are born with a Life Limiting Fetal Diagnosis.	No applicable	Integrative literature review
S9^20^	USA 2020	Synthesize parental decisions through potentially life-limiting fetal diagnosis in pregnancy and the principles of perinatal palliative care	Not applicable	Literature review
S10^21^	Australia 2020	Provide empirical Australian evidence of views and experiences of care provision from health professionals (HPs) and parents	11 parents (7 mothers and 4 fathers, from 7 parental couples), who decide to continue a pregnancy following a diagnosis of a lethal fetal abnormality and 08 professionals	Qualitative phenomenological study
S11^22^	USA 2020	Review recent and relevant literature on the importance of birth planning and the role of perinatal palliative care when a life-limiting fetal diagnosis is made	Not applicable	Literature Review
S12^23^	Ireland 2019	Increase the understanding of the lived experience of mothers with a prenatal lethal diagnosis who continued their pregnancy, and their response to the care they received.	4 women/mothers	Qualitative phenomenological study
S13^24^	USA 2019	Discover important components of a birth plan for parents and providers who carry them out, and understand the experience of parents and providers with birth plans.	20 parents living a pregnancy complicated by a life-limiting diagnosis and 116 providers	Mixed-methods, descriptive, exploratory survey
S14^25^	USA 2018	Describe parents’ experiences of continuing a pregnancy with an LFD and to examine parents’ needs and responses to healthcare provider interactions in the prenatal, intrapartum, and postnatal periods.	30 parents (16 mothers and 14 male partners)	Qualitative phenomenological study
S15^26^	USA 2017	Describe, using literature on trisomy 13 and trisomy 18, how information shared between parents and providers can improve perinatal counseling and family support.	Not applicable	Literature review
S16^27^	Ireland 2017	Explore parents’ perception of pregnancy and the loss of a twin, their experience of diagnosing a congenital abnormality and their experience of CPP.	09 parents (5 women/mothers and 4 men/fathers)	Qualitative phenomenological study
S17^28^	USA 2016	Investigate the developmental tasks of pregnancy that parents undertake after receiving a lethal diagnosis and continuing the pregnancy.	16 mothers, 13 fathers and 1 female partner.	Qualitative phenomenological study
S18^29^	Canada 2016	Examine parental goals/decisions, the length of life of their child and factors associated with survival.	332 parents (202 men/fathers and 59 women/mothers)	Descriptive quantitative study
S19^30^	USA 2016	Share the experience of having an infant in the NICU, perspectives of a life transformation and lesson in the hope of helping health professionals consider a balanced view of the NICU’s impact on families.	25 health professionals parents or relatives of high-risk newborn	Experience report
S20^31^	New Zealand 2013	Describe the journey for a family when they decided to continue the pregnancy and build a support network to meet their personal, physical, emotional, cultural, religious and spiritual needs.	One case	Case study
S21^32^	USA 2013	Synthesize the existing qualitative literature about parental involvement in ethical decision making for critically ill neonates and develop a preliminary theoretical framework of parent ethical decision making.	Not applicable	Qualitative meta-ethnography
S22^33^	USA 2012	Report findings related to the varying notions of hope between parents who were at risk of a periviable delivery and their healthcare professionals.	40 women/mothers and 14 male partners/fathers	Qualitative narrative study
S23^34^	USA 2011	Describe how parents make life support decisions for extremely premature infants from the prenatal period through death from the perspectives of parents, nurses, and physicians.	5 cases involving life support decisions.	Case study
S24^35^	USA 2011	Describe the experiences of women and male partners as they interpreted and made meaning of the fact that their fetus was substantially impaired, with particular attention to the way facts were presented to them and how they constructed their own truth.	15 women and 10 of their male partners	Qualitative ethnography study
S25^36^	USA 2011	Increase practitioners’ awareness of spiritual and existential distress and to provide strategies to address such needs, particularly at the end of life.	6 cases	Case study
S26^37^	USA 2011	Explore parents’ experience with a lethal fetal diagnosis during pregnancy to gain insight into their needs.	5 women/mothers and 3 men/fathers; 3 couples took part in the study together.	Qualitative descriptive study
S27^38^	USA 2005	Examine the experience of low-income, African-American parents surrounding perinatal loss and to describe how other life stressors influenced the parents’ responses and caring needs.	23 parents, from 17 families consisting of 17 mothers and 6 of their male partners.	Qualitative phenomenological study

**Table 2:** Main hope dimensions and hope evidence in parental experience of perinatal and neonatal palliative care

Hope Dimensions	Hope evidence
**Cognitive**	Sense of control, identity, normality and validation for themselves and their baby (S1) [12] Parents hope that the diagnosis could be wrong or hope that it is not as bad as predicted (S5) [13] Information is vital (S12) [14]; (S20) [15]; (S22) [16]; (S23) [17]. To obtain information using Internet was distressing (S12) [14]; (S23) [17] Parents to express their goals, fears, hope help them do stablish decisions (S15) [18]; (S17) [19]; S20 [15] and emotions (S27) [20] Parents hope after the diagnosis is designed for: meet their child alive, spend some time as a family, bring their child home, and give their child a good life. Parents wanted to give them a chance (S18) [21] Describe the process as a life transformation, in which the need for hope, honesty in prognosis, compassion and facilitation of connection (S19) [22] The mothers need make plans for the birth of their baby, and to cope later in the Neonatal Intensive Care Unit (S21) [23] Finding meaning is one of the first step for parents to begin to reorganize their lives (S25) [24] Parents avoid being given false hope. False hope is about information too optimistic given to make parents feel better. (S20) [15]
**Affective**	Parents belief that the child is alive, a chance for him to fight could be given, and miracles could occur (S2) [25]; (S7) [26]; (S10) [27]; (S15) [18]; (S23) [17]; (S24) [28]; (S27) [20] Parents` decisions about continuing or interrupting the pregnancy is linked to their beliefs, and meaning gave to child existence (S3) [29]; (S6) [30]; (S8) [27]; (S17) [19] Parents share a theme of discerning what makes for a valued life, highlighting their quality-of-life considerations for the unborn child and existing family members (S5) [13]; (S11) [31] This perception of themselves as a mother and the bond with their baby remained (S6) [30] Families rely on their faith and spirituality in times of crisis to help them make sense of the path to the end of life (S8 [32]; S20 [15]) Parents often have an intrinsic need to feel that they have “done the best that they could” for their child (S9) [33] The couple’s decision was to normalize and celebrate the pregnancy and the child life (S20) [15]; (S26) [34] They want counseling without judgment, with attention to continuity of treatment and a hopeful approach that recognizes the uncertainty of prognosis (S26) [34] The transformations of life involve changes of perspectives, acceptance of uncertainty e lack of control, advances in gratitude, humility, valuing emotions e feelings and forgiveness. (S19) [22] They value connections, belief, and possibilities of rewriting stories. They became more compassionate and hopeful as people and providers. (S19) [22]
**Temporal**	The parents struggled through the situation as a couple and envisioned what the future would bring (S2) [25] Memorabilia and clinical milestones in their pregnancies validated these maternal identities (S6) [30] All mothers learned to focus on living in the present (S12) [14] When a life-limiting diagnosis is made, the remainder of the pregnancy and time afterward are vastly different than what was anticipated and hoped for (S13) [35] Decision to enjoy the pregnancy despite limit time of the time life of their baby (S16) [36]
**Affiliative**	Connectedness to others (children, family, friends and other parents) played a part in parents’ resilience and emotional wellbeing (S1) [12] (S26) [34] Sharing this decision with their family and social network helped them hope. (S20) [15] (S23) [17] Contacting experience of parents in similar situations was helpful (S20) [15] When pregnancy became obvious mothers felt difficulties with social relations but engaged with mothers who had a similar experience. (S12) [14] To attach with the child during his existence is hopeful, as well as being invited to think about willing to establish memories. (S3) [29] Fetal movement helped a woman to replace sadness and to feel more affection for the child. (S26) [34] To invest in the relation with the survival twin helped parents to hope and suffer less. (S16) [36] Fathers often kept their emotions under control for fear of further upsetting the mother but were also unsure how to support their partners. (S27) [20] Spirituality and religion assisted their progression and feelings of hope for their future without their child. (S1) [12] and helped them to make sense of the situation. (S4) [37] Parents need collaborative discussions with specialists to make their decisions. (S10) [27] Feeling part of the team and being able to discuss things openly with them helped to make decisions. (S2) [25]; (S20) [15]; (S23) [17]. Feeling freely included and involved was experienced in a positive way (S21) [23], balancing autonomy and parental authority with medical recommendations and prognosis. (S11) [31]; (S24) [28] Feeling that professionals are compassed, available, honest and empathic contributes to making decisions and to hope and comfort (S3) [29]; (S8) [32]; (S14) [38]; S22 [16] To feel involved, have a parental role, and have their wishes respected (S13) [35]. In the other hand, lack of sensitive attunement from the providers intensified the trauma. (S12) [14];(S21) [23]; (S22) [16]; (S24) [28] To balance moments of hope and hopelessness is complex and is linked with professionals’ attitudes and the information given by providers (S14) [38] Hope is promoted through valuing and allowing parents needs in terms of interacting with their child. (S14) [38] Parents recognized nurses’ efforts for “special attention” regards to their physical, emotional, and spiritual needs, as helpful. (S27) [20]

## Results

The updated research retrieved 1.341 studies; 64 were excluded due to duplication, and 1,277 had their titles and abstracts read. In turn, 1,229 were excluded, leaving a total of 48 articles: 3 of them were not accessible, and 45 were read in full. Of these articles, 18 were excluded for not meeting the established inclusion criteria (17 did not focus on the parental hope experience, and 1 was a brief book review), leaving the final 27 articles (Fig. 1), identified with the letter ‘S’ in the study reference and an Arabic number, for example: ‘S1’ for Study 1 (Table 1).

Regarding year of publication, country and method, this review found that there are mainly articles published in the last five years (n = 13), carried out in the United States of America – USA (n = 15), and structured as phenomenological reviews (n = 7) and literature reviews (n = 7) (Table 1).

The mapping showed that the scientific production on the subject matter of parental hope in perinatal and neonatal palliative care is recent, covering 18 years, with the first study published in 2005 and a more significant production trend from 2011 onwards, maintaining certain homogeneity and consistency from 2018 onwards, indicating an incipient and emerging knowledge area.

The characteristics of the participants included in the studies revealed centrality of women’s perspectives in the context of pregnancy and perinatal palliative care.

The hope dimensions proposed by Dufault and Martochio [5] were identified in the studies under review. The ones that had the greatest expression were Cognitive, Affiliative, Affective and Temporal (Table 2)

## Discussion

Faced with the diagnosis and initial information about the child’s situation, the parents’ hope process has been linked to the management of “uncertainties” and the revelations of “certainties”, when information support is crucial and linked to parent-professional interactions [25–29, 13–36, 21–24]. Parents need collaborative discussions with specialist providers to make decisions about the continuity of the pregnancy and childcare [23, 27, 28]. Trust must be nurtured in care relations because it influences hope and decision-making [23]. In sum, information support exerts effects mainly in the affiliative and cognitive hope dimensions. In this way, parents can identify goals they want to achieve in the short and medium term, as they have the necessary and realistic information to support their experiential processes.

In most cases, in the peri/neonatal PC context, the diagnosis is made by an ultrasonographer who is not part of the family’s care and, therefore, has no knowledge of the family’s life history on first contact. When communication is carried out in a hostile manner and in an unwelcoming environment and ambience, it becomes devastating for hope [17–28], again highlighting the cognitive and affiliative hope dimensions.

The articles included in this scope point to the need for parents to process the information they receive to gradually establish attachment and bonds, value time with their newborn, create memories and experience parenthood [12, 29, 30, 27–38, 15–23, 17–28], which is strongly connected to the affiliative, affective, and temporal hope dimensions. However, there is interference in the cognitive and behavioral dimensions, as it is under the understanding that time with the child is limited and uncertain (temporal and affiliative dimensions) that the parents expressed the desire to make the most of the pregnancy moments, such as naming the child, which lends value to the newborn’s humanity; including it in family rituals (having a Baby Shower), celebrations and holidays; seeking closer physical, auditory and visual interactions with it before and after birth; setting up the baby’s room and buying layette items; going on walks and trips for the newborn to visit different places; and experiencing and performing activities such as other pregnant women (behavioral dimension). These are attachment and care actions with the child [37, 27, 18–36] (affiliative dimension).

Social support takes part in feelings of hope, well-being and resilience and is singular in terms of who they want to connect to [12, 15, 28, 31]. On the other hand, religion and spirituality were highlighted in the articles as important to make sense of the situation and distress faced [13, 32, 33, 31, 24]. Spiritual and religious beliefs contribute to understanding life events and are related to the affiliative dimension of hope. Through religion and spirituality, people seek to mitigate the agony of the finitude of life and their suffering. For parents who have decided to invest in their child’s life, hope is sustained by the belief that life is important; therefore, the child deserves a chance to live [25–29, 13–30, 32, 27–31, 19, 15], as well as by the understanding that having time with the newborn is an opportunity for parenthood [13–31, 19, 15, 34]. Higher power and spiritual beliefs guided hope in some studies [24, 31, 32]. The above illustrates that the affective and affiliative hope dimensions have a close connection.

The quality and nature of the relationships involved in parental care shape the parents’ needs, promote a positive experience and a parental role, deal with emotions and resilience [12, 20, 23, 35], and have their wishes respected [35]. This articulation illustrates the relation between the affiliative, affective and cognitive hope dimensions.

The interaction with the professionals at the time of the diagnosis or suspicion was highlighted in the review, attributed as a distressing and unwelcoming moment related to hopelessness. The parenting process is taking place; mothers and fathers are facing a complex and difficult time, in which hope is an imperative of parental existence [39]. Information about the diagnosis or its suspicion interferes with the newborn’s existence in family life (cognitive dimension); therefore, the way in which professionals inform and support bereavement is crucial.

The expansion of prenatal screening and technological advances tends to amplify such situations and, therefore, the establishment of interactions directed to the parents’ needs, focusing efforts on the people affected by the news, respecting silences, providing opportunities for conversations, and placing them at the center of the care provided.

This review identified health professionals’ actions as a direct determinant of parents’ hopes and, therefore, of their coping and the scope of care and its alignment with PC precepts. However, a tendency has been described for professionals to quickly diagnose perinatal conditions that determine the need for PC, without talking to parents about the meaning of such diagnosis and its consequences, let alone sharing decisions with them [40]. The professionals’ effective communication skills and preparedness to work in PC cases are extremely important and linked to the parents’ hope and quality of their experience in PC [40]. However, professionals are currently unprepared to deal with the vulnerabilities, demands and needs that emerge in the PC context [41–43], which is seen in the results of this review. It would be a contribution if health professionals’ training turned its gaze to human subjectivity and incorporated this discussion, expanding a human and singular view of care.

This scoping review has produced a broad mapping and innovative synthesis of evidence of parental hope in perinatal and neonatal palliative care. The limitations of this study are the languages (English, Portuguese, and Spanish) selected and the noninclusion of gray literature.

### Implications of the findings for practice

The consideration of hope is a challenge for professionals in the context of caring for parents under perinatal and neonatal PC. Recognizing manifestations of hope-hopelessness creates, invests, and maintains a context that supports emotional, collaborative, and therapeutic relationships.

Adopting a shared care practice with availability to informative translation, concerning the parents’ time processing to make their decisions, as well as timely and impact communication and sharing decision-making, can enable control and support families to accept the reality of their situation while maintaining a sense of hope in its broadest sense.

Collaboratively establishing a birth plan supports the parental hope experience, especially in determining hope objects. The development of perinatal and neonatal palliative care programs should be structured in the framework of family-centered care in the hope experience.

Adopting evidence of hope in perinatal and neonatal palliative care centered on parents and families is a way of transforming clinical practice with a focus on reaffirming life and creating positive memories and experiences, which is directly related to parental and family empowerment and resilience.

### Implications of the findings for research

This review reveals gaps and points to the need for studies that explore fathers’ hope experience and the relationship with decision-making as parents. The same is true at the family level. It is important to consider the diversity of types of families and parenthood, such as homosexual couples and parents, which were only addressed in one of the studies included in this review.

It is possible to gain a greater understanding of the relationship between parental autonomy and hope. The temporal, behavioral and contextual hope dimensions in the parents’ experience are insufficiently explored and comprehended in the perinatal and neonatal PC context.

Qualitative studies are needed to gain a deeper understanding of how the process of “hoping” takes place in this population during the perinatal and neonatal periods. Once this phenomenon is better understood, studies could be carried out using mixed research methodologies, which would allow the “promoting hope” intervention to be evaluated in terms of health gains for this population.

## Conclusions

This review asserted hope as an important coping mechanism for parents in the context of perinatal and neonatal PC. When there was a prevalence of uncertainties, hope supported the belief in a potentially different outcome from the one predicted by the professionals, and in the face of confirmations, it turned to the exercise and expression of parenting through the intentional creation of bonds, care actions and memories. Hope is a constant in the experience of parents living with the diagnosis of a fetal condition compromising the child’s life. It contributes to the research on movement and rebalancing, acting as a driving force and with a tendency to push parents from inertia.

Hope is the strength that guides parents in facing uncertainties through the diagnosis of a condition that compromises their child’s life. Interactions with health professionals exert a direct impact on hope and, consequently, on family behaviors and responses. Parents believe that health professionals can manage the family’s hope by establishing “sensitive” therapeutic relationships that focus on the therapeutic dimension of hope: assertive communication, transmitting clear, realistic, and respectful information. Hope positions parents on a coping line consonant with their beliefs and reality. This experience contributes to comfort, trust in professionals and confidence in decision-making, serving as a guide and autonomy booster. The relational contexts (especially with professionals) in palliative care suggest inadequacies in promoting the parents’ hope experience.

Accessing and recognizing hope dynamics is a premise for health care aimed at quality of life, comfort, and autonomy for parents in perinatal and neonatal PC, and therefore, it is a professional commitment.

Diverse evidence of parental hope has been mapped in this review in light of its dimensions, with contributions to assessing and identifying hope objects, resources and threats.

### Electronic supplementary material

Below is the link to the electronic supplementary material.

Supplementary Table 1: Full search strategy for each database

## Data Availability

All data and materials analyzed during this study are included in this manuscript.
