# Supplementary material for: Parents’ hope in perinatal and neonatal palliative care: a scoping review
Source: BMC Palliat Care. 2023 Dec 18;22:202. doi: 10.1186/s12904-023-01324-z (PMC10726497; doi:10.1186/s12904-023-01324-z)
Supplement: Supplementary file 1 — Supplementary Table 1: Full search strategy for each database [file 12904_2023_1324_MOESM1_ESM.docx]

Supplementary Table 1. Full search strategy for each database.

| Database | Number of articles | Search terms |
| --- | --- | --- |
| MEDLINE | 398 | “hope"[MeSH Terms] OR "hope"[All Fields]) AND "famil*"[All Fields] AND ("mothers"[All Fields] OR "mothered"[All Fields] OR "mothers"[MeSH Terms] OR "mothers"[All Fields] OR "mother"[All Fields] OR "mothering"[All Fields]) AND ("father s"[All Fields] OR "fathered"[All Fields] OR "fathers"[MeSH Terms] OR "fathers"[All Fields] OR "father"[All Fields] OR "fathering"[All Fields]) AND ("infant, newborn"[MeSH Terms] OR ("infant"[All Fields] AND "newborn"[All Fields]) OR "newborn infant"[All Fields] OR "neonatal"[All Fields] OR "neonate"[All Fields] OR "neonates"[All Fields] OR "neonatality"[All Fields] OR "neonatals"[All Fields] OR "neonate s"[All Fields])) OR ("perinatal"[All Fields] OR "perinatally"[All Fields] OR "perinatals"[All Fields])) AND ("palliative care"[MeSH Terms] OR ("palliative"[All Fields] AND "care"[All Fields]) OR "palliative care"[All Fields] OR "palliative"[All Fields] OR "palliatively"[All Fields] OR "palliatives"[All Fields])) NOT "oncolog*"[All Fields]) NOT "genetic*"[All Fields]. |
| CINAHL | 720 | Hope AND Palliative care AND Perinatal OR Neonatal AND Parent$ OR Famil$. |
| PsycINFO | 223 | “hope"[MeSH Terms] OR "hope"[All Fields]) AND "famil*"[All Fields] AND ("mothers"[All Fields] OR "mothered"[All Fields] OR "mothers"[MeSH Terms] OR "mothers"[All Fields] OR "mother"[All Fields] OR "mothering"[All Fields]) AND ("father s"[All Fields] OR "fathered"[All Fields] OR "fathers"[MeSH Terms] OR "fathers"[All Fields] OR "father"[All Fields] OR "fathering"[All Fields]) AND ("infant, newborn"[MeSH Terms] OR ("infant"[All Fields] AND "newborn"[All Fields]) OR "newborn infant"[All Fields] OR "neonatal"[All Fields] OR "neonate"[All Fields] OR "neonates"[All Fields] OR "neonatality"[All Fields] OR "neonatals"[All Fields] OR "neonate s"[All Fields])) OR ("perinatal"[All Fields] OR "perinatally"[All Fields] OR "perinatals"[All Fields])) AND ("palliative care"[MeSH Terms] OR ("palliative"[All Fields] AND "care"[All Fields]) OR "palliative care"[All Fields] OR "palliative"[All Fields] OR "palliatively"[All Fields] OR "palliatives"[All Fields])) NOT "oncolog*"[All Fields]) NOT "genetic*"[All Fields]. |
